# Testing for Differences in Metabolism Among Females and Dimorphic Males of Four Dung Beetle Species (Coloeoptera: Scarabaeinae)

**DOI:** 10.1093/iob/obaf031

**Published:** 2025-07-26

**Authors:** A T Killeffer, J M Fleming, A Padukone, N Duerr, K A Reed, J Merizalde-Toro, K E Marshall, J E Celi, K S Sheldon

**Affiliations:** Department of Ecology & Evolutionary Biology, University of Tennessee, Knoxville, TN 37996, USA; Department of Ecology & Evolutionary Biology, University of Tennessee, Knoxville, TN 37996, USA; Department of Ecology & Evolutionary Biology, University of Tennessee, Knoxville, TN 37996, USA; Department of Ecology & Evolutionary Biology, University of Tennessee, Knoxville, TN 37996, USA; Department of Ecology & Evolutionary Biology, University of Tennessee, Knoxville, TN 37996, USA; Water and Aquatic Resources Research Group, Universidad Regional Amazónica Ikiam, Tena 150158, Ecuador; Department of Zoology, University of British Columbia, Vancouver, BC V6T 1Z4, Canada; Water and Aquatic Resources Research Group, Universidad Regional Amazónica Ikiam, Tena 150158, Ecuador; Department of Ecology & Evolutionary Biology, University of Tennessee, Knoxville, TN 37996, USA

## Abstract

Both sexual and male dimorphism are common in nature, yet we have limited understanding of how different developmental pathways and reproductive strategies of morphs shape energetics. To address this gap, we examined metabolic rates of four species of dung beetle (*Onthophagus taurus, Onthophagus hecate, Oxysternon silenus*, and *Phanaeus vindex*) with both sexual and male dimorphism. In these species, males have horn length dimorphism, including larger-horned (“major”) males and smaller-horned (“minor”) males. The gene doublesex, *dsx*, drives both sexual dimorphism and, by mediating nutrition-dependent horn growth in some species, male dimorphism. Because females and minor males share developmental pathways and have greater investment in reproductive organs than major males, we hypothesized energetic costs would be similar and higher in females and minor males compared to major males. To test this hypothesis, we examined metabolic rates of morphs using flow-through respirometry to record CO_2_ output. After accounting for body size and activity level, we found that in two species, *Onthophagus taurus* and *Phanaeus vindex*, females had higher CO_2_ production compared to major males, and in *O. taurus*, females also had higher CO_2_ production than minor males. We detected no differences between sexes for *O. hecate* and *O. silenus*. We also found no significant difference in metabolic rates between major and minor males of any species. Our results suggest that, for these species of dung beetles, any energetic tradeoffs due to reproductive strategies occur between females and males, but not between male morphs. The lack of a general trend in metabolic rates suggests energetic costs are decoupled from sex and male morph across dung beetle species, which runs counter to evolutionary explanations for the maintenance of alternative reproductive tactics.

## Resumen

Tanto el dimorfismo sexual como el dimorfismo entre machos son comunes en la naturaleza, sin embargo, tenemos un conocimiento limitado de cómo las diferentes vías de desarrollo y estrategias reproductivas de los morfos alteran la dinámica energética. Para abordar esta brecha, examinamos las tasas metabólicas de cuatro especies de escarabajos estercoleros que presentan tanto dimorfismo sexual como dimorfismo entre machos (*Onthophagus taurus, Onthophagus hecate, Oxysternon silenus* y *Phanaeus vindex*). En estas especies, los machos tienen dimorfismo en la longitud de los cuernos, incluyendo machos de cuernos grandes (“mayores”) y machos de cuernos pequeños (“menores”). El gen de doble sexo, dsx, impulsa tanto el dimorfismo sexual como, al mediar el crecimiento de los cuernos dependiente de la nutrición en algunas especies, el dimorfismo masculino. Dado que las hembras y los machos menores comparten vías de desarrollo e invierten más energía en sus órganos reproductivos que los machos mayores, planteamos la hipótesis de que los costos energéticos serían similares entre hembras y machos menores y más altos que en los machos mayores. Para probar esta hipótesis, examinamos las tasas metabólicas de los morfos utilizando la respirometría de flujo para registrar la producción de CO2. Después de tener en cuenta el tamaño corporal y el nivel de actividad, encontramos que en dos especies, *O. taurus* y *P. vindex*, las hembras tuvieron mayor producción de CO_2_ que los machos mayores, y que las hembras de *O. taurus* también tenían una mayor producción de CO_2_ que los machos menores. No detectamos diferencias significativas entre sexos para *O. hecate* y *O. silenus.* Tampoco encontramos diferencias en las tasas metabólicas entre los morfos mayores y menores de ninguna especie. Nuestros resultados sugieren que, en estas especies de escarabajos estercoleros, los posibles compromisos energéticos debido a las estrategias reproductivas ocurren entre hembras y machos, pero no entre morfos masculinos. La falta de una tendencia general en las tasas metabólicas sugiere que los costos energéticos están desvinculados del sexo y la morfología masculina en todas las especies de escarabajos estercoleros, lo que va en contra de las explicaciones evolutivas para el mantenimiento de tácticas reproductivas alternativas.

## Introduction

Selective pressures associated with reproduction shape the development and physiology of males and females and can also lead to phenotypic variation within a sex. A well-documented example of this is male dimorphism in the size of traits, such as horns or claws, which reflects alternative reproductive tactics; larger, more weaponized (“major”) morphs tend to guard females and smaller (“minor”) morphs use non-fighting and sneaking tactics to gain access to females (Gross 1996; Oliveira et al. 2008). In some species, the developmental mechanisms leading to male trait dimorphism also regulate sexual dimorphism (Emlen et al. 2005). Consequently, phenotypic traits of some male morphs may be more similar to females than other male morphs of the species. Despite the frequency of male dimorphism in nature (Oliveira et al. 2008), little is known about physiological variation among females and dimorphic males (Shingleton and Vea 2023). In this study, we focus on four species of dung beetle (Coleoptera: Scarabaeinae) with males showing horn length dimorphism to understand how sex- and male morph-specific developmental trajectories and reproductive strategies impact adult metabolic rates.

In dung beetles, shared developmental pathways suggest that the energetics of minor males may resemble females rather than major males. During dung beetle development, the mechanisms regulating sexual dimorphism also generate male dimorphism in horn length. Studies show that in insects, the gene *doublesex* (*dsx*), which has a highly conserved structure and function, regulates the expression of downstream genes that drive sexually dimorphic development (Sánchez 2008; Shukla and Nagaraju 2010a; Williams and Carroll 2009; Ledón-Rettig et al. 2017). The expression of sex-specific *dsx* transcription factors occurs due to alternative splicing of an exon that is included in *dsx* transcripts in males and excluded in females (Shukla and Nagaraju 2010b; Wexler et al. 2019). In some dung beetles in the genus *Onthophagus, dsx* also regulates male dimorphism in horn length (Kijimoto et al. 2012). For example, in the dung beetle *Onthophagus taurus, dsx* expression results in variation within males by mediating nutrition-dependent horn growth, with minor males resulting from reduced larval provisioning compared to major males (Emlen and Nijhout 1999; Kijimoto et al. 2012). In the dung beetle *Digitonthophagus gazella, dsx* mediates sexual size dimorphism; knocking down *dsx* increased female size and decreased male size (Rohner 2021). This suggests that by impacting male body size, *dsx* could influence intraspecific variation in life history among male *D. gazella*. Thus, the same genetic machinery can drive sex- and morph-specific development of horns in some dung beetles (Kijimoto et al. 2012); not surprisingly, research shows that when horns are present in females, they also tend to be present in minor males (Emlen et al. 2005). Importantly, *dsx*, which causes anatomical sexual dimorphism, can also impact sex differences in metabolism (Coig et al. 2024). However, as an autosomal gene, the effects of *dsx* on sex-specific metabolism can be decoupled from the effects of sex chromosomes (Coig et al. 2024). Because sexual and male dimorphism have not evolved independently in dung beetles, the shared developmental pathways of females and minor males may result in similar energetic phenotypes.

In addition to developmental mechanisms, selective pressures associated with reproduction suggest the energetics of minor males are more similar to females than major males. Different reproductive strategies require different types of investment. In dung beetles, major males invest in large horns that have costs both during and after development. During development, the large horns come at the cost of an extended larval period, increased mortality, and limits on the allocation of resources to other morphological traits (Hunt and Simmons 1997; Nijhout and Emlen 1998; Moczek and Emlen 2000). As adults, major males engage in energetically costly behaviors; they use the large horns to both compete directly for mating opportunities and actively guard females. In contrast, minor males utilize a sneak strategy to copulate, but they have fewer mating opportunities, and their sperm is often subjected to post-copulatory competition from major males (Moczek and Emlen 2000). Compared to major males, minor males show greater investment in reproductive organs, including larger testes size and higher sperm quality, which may give an advantage during sperm competition (Parrett et al. 2022; Simmons et al. 1999, 2007). Females mirror minor males in having fewer mating opportunities and investing more in reproductive organs compared to major males (Rönn et al. 2006; Hayward and Gillooly 2011). This dichotomy of reproductive strategies may be reflected in energetics.

Metabolic rates vary by sex and may also vary in dimorphic males. As adults, female and male insects have different energetic demands, yet there is no clear trend in metabolic rates; depending on the species, both females (DeVries et al. 2014; Shingleton and Vea 2023) and males (Tomlinson and Phillips 2015) have displayed higher metabolic rates. These differences may reflect the different developmental pathways and unique energetic requirements related to gamete production (Shingleton and Vea 2023) or differences in behavior associated with reproduction (Cummings and Gelineau-Kattner 2009). However, minor males that share developmental pathways and reproductive strategies with females could have metabolic phenotypes more similar to females than major males (Shingleton and Vea 2023). Despite much prior work on dung beetle metabolism (e.g., Davis et al. 1999; Terblanche et al. 2010; Sheldon et al. 2020), little is currently known about the role that male dimorphism plays in metabolic rates.

To understand how energetics vary among beetles with both sexual and male dimorphism, we measured the metabolic rates of female and major and minor males of four species of tunneling dung beetle: two species (*Onthophagus taurus* and *Onthophagus hecate*) from the tribe Onthophagini Streubel, 1846, and two species (*Oxysternon silenus* and *Phanaeus vindex*) from the tribe Phanaeini Hope, 1838. Tunnelers breed by arriving at dung pats, digging tunnels nearby, and packing dung into tunnels to form a brood mass (Moczek and Emlen 2000; Price and May 2009). An egg is then laid in the brood mass, and when the egg hatches, the larva feeds only on dung provided in the brood mass. Horn formation in males is primarily driven by larval nutrition, with major males developing from better nutrition (Emlen 1994). For this reason, the body size of minor males tends to be smaller than both major males and females. Previous work on dung beetles shows that, when mating, the major morphs with large horns and overall larger body size tend to guard females through head-to-head combat (Rasmussen 1994; Moczek and Emlen 2000). Smaller, minor morphs use non-fighting and sneaking tactics to gain access to females. When major males observe minor males copulating with a female, major males mate with females again (Moczek and Emlen 2000). Thus, the biological fitness of major males benefits most from pre- and post-copulatory investment, and fitness of minor males benefits primarily from post-copulatory investment (Hunt and Simmons 2001; Simmons and Buzatto 2014).

Though much prior work has compared sex differences in metabolic rates, few studies have tested for differences in metabolism both between sexes and within a sex. Because dung beetle females and minor males have similar developmental pathways and both invest in reproductive organs that are energetically costly to maintain, we hypothesized that energetic costs would be similar and higher in these two morphs compared to major males. Thus, we predicted metabolic rates would be higher in minor males and females compared to major males. Alternatively, major males that engage in aggressive mating tactics of direct competition and mate guarding may display greater metabolic rates compared to females and minor males.

## Methods

### Study species

We examined the metabolic rates of four species of tunneling dung beetle (*Onthophagus taurus, Onthophagus hecate, Oxysternon silenus, and Phanaeus vindex*) with both sexual and male dimorphism (Fig. S1).

### Onthophagus hecate

*Onthophagus hecate* occurs across North America, ranging from Florida to southern Canada (Hoebeke and Beucke 1997). Major and minor males are differentiated by the presence or absence, respectively, of a third downward-pointing tooth on an otherwise bifurcated pronotal horn (Nemes and Price 2015). In addition, minor males have a reduced to highly reduced pronotal horn, and major males have a sub-triangular tooth on the clypeus. Females have two transverse ridges on the head.

### Onthophagus taurus

*Onthophagus taurus* are native to Europe and were first found in the United States in the mid-1970s on cattle pastures in Florida (Fincher and Woodruff 1975; Hoebeke and Beucke 1997). The species has since spread northward into much of the eastern and southeastern United States and southward into the Caribbean (Floate et al. 2017; Pokhrel et al. 2021). Major males of the species are distinguished by their possession of two long, curved horns extending from the head across the pronotum. In minor males, two small tubercles protrude from the base of the head in place of horns. Females possess no horns and two transverse ridges on the head (Nemes and Price 2015).

### Phanaeus vindex

*Phanaeus vindex* occurs east of the North American continental divide from the upper east coast to Florida and northern Mexico (Edmonds 1994). Males are differentiated from females by the presence of a single horn of variable length on their heads (Nemes and Price 2015). The pronotum of major males is a flat, sub-triangular shield with acute, elevated, posterior ridges. Minor males lack a shield, and if posterior ridges are present, they are neither elevated nor acute. Females possess a single transverse tubercle (Nemes and Price 2015).

### Oxysternon silenus

*Oxysternon silenus* is widely distributed across northern and northwestern South America into Central America (Edmonds and Zedík 2004). Major males are distinguished by a long, erect, cylindrical head horn and a pair of upward-pointing pronotal spines. Minor males lack a horn with a bidentate prominence in its place and have reduced perinatal spines. Females have a strongly raised trituberculate ridge separating the clypeus and frons (Edmonds and Zedík 2004).

### Field collection

We used baited pitfall traps to collect females, major males, and minor males. We set traps in the late morning and collected beetles within 4–12 h. We collected *O. silenus* (June–July 2022) near Tena, Ecuador (0°57′′02′′S, 77°51′′54′′W; permit no. MAATE-DBI-CM-2021–0177 to JEC), *P. vindex* (July–August 2021) and *O. hecate* (September 2022) at Seven Island State Park in Kodak, Tennessee (35°57′′143′′N, −83°41′′221′′W; permit no. TDEC-2020–030 to KSS), and *O. taurus* (September 2022) on private property in Clinton, Tennessee (36°09′07.0°N, 84°06′30.3°W). We transported beetles to the lab in 950 ml plastic containers with moist paper towels. We housed beetles in the lab in colonies separated by species and sex at room temperature (22°C) and fed them cow dung *ad libitum* prior to acclimation. We held beetles in these colonies for ∼7–14 days prior to acclimation treatments, and used field-caught individuals for our experiments.

Metabolic rates can vary with age in some beetle species, particularly just following emergence and close to winter diapause (Piiroinen et al. 2010). Though we were not able to account for the age of the beetles, the morphs of each species were randomly caught over the same 4–8 week period, the beetles were not teneral (i.e., newly emerged), and experiments were conducted well before overwintering. For these reasons, we do not expect age to be driving the results we found.

### Acclimation treatments

For acclimation, we placed a single, field-caught beetle in either a 74 mL plastic container (*O. taurus, O. hecate*) or a 177 mL plastic container (*O. silenus, P. vindex*) filled with a moist soil mixture (4:1 parts soil: sand) and fed the beetles cow dung *ad libitum*. We placed the containers with beetles into incubators (PHCbi model MIR-554-PA) at 22 ± 2°C (12:12 L:D) for 6 days. We checked beetles every 2–3 days, replaced the cow dung, and maintained soil moisture.

### Metabolic rate

To record metabolic rates of each morph (54 females, 66 minor males, and 69 major males across the four species; Table 1), we used flow-through respirometry with the Q-Box RP1LP Low Range Respiration Package from Qubit Systems. We set the flow rate to 80 mL/min for *O. taurus* and *O. hecate* and 100 mL/min for *O. silenus* and *P. vindex*. For our air source, we used zero air scrubbed of carbon dioxide (CO_2_) using Ascarite^TM^ and water (H_2_O) using Drierite drying desiccant. We measured the CO_2_ production of each beetle at 15°C. We used this test temperature because we wanted to measure metabolic rates as close to resting as possible, and beetles are less active at 15°C compared to 22°C. We randomly chose beetles and assigned them to either morning or afternoon trials.

**Table 1:** Summary statistics of dung beetle species by morph, including sample size, beetle mass (measured before metabolic trials), mean CO_2_ production, and activity by morph.

Species	Morph	*n*	Initial mass, mean (SD), g	CO_2_ production, group mean (SD), mL min^−1^	Mean activity (SD)
*O. hecate*	female	18	0.052 (0.01)	0.0149 (0.0034)	1.89 (0.32)
	major	20	0.057 (0.009)	0.0159 (0.004)	1.75 (0.44)
	minor	20	0.044 (0.004)	0.0139 (0.0038)	1.8 (0.41)
*O. silenus*	female	20	0.607 (0.078)	0.0421 (0.0097)	1.5 (0.51)
	major	17	0.591 (0.049)	0.0435 (0.0068)	1.47 (0.47)
	minor	11	0.434 (0.06)	0.0335 (0.0083)	1.01 (0.04)
*O. taurus*	female	10	0.089 (0.021)	0.0257 (0.0082)	1.46 (0.48)
	major	20	0.079 (0.013)	0.019 (0.0043)	1.95 (0.22)
	minor	19	0.065 (0.008)	0.019 (0.0039)	1.83 (0.36)
*P. vindex*	female	6	0.851 (0.179)	0.0637 (0.0097)	1.83 (0.41)
	major	12	0.784 (0.093)	0.0453 (0.0113)	1.88 (0.54)
	minor	16	0.544 (0.052)	0.0451 (0.0075)	1.95 (0.41)

At the start of a trial, we removed the beetle from the acclimation incubator, recorded mass (Mettler Toledo model ML54T, 0.1 mg accuracy), placed the beetle into a 15-mL glass chamber connected to the respirometry equipment, and then put the chamber into a different incubator (RevSci Incufridge) set to 15°C. We held the beetles there for 15 min; the first 10 min served to acclimate the beetles to the trial, and the following 5 min served as the recording window where we measured CO_2_ production. During the recording window, we used a camera probe with a small light (Teslong NTS500B 5mm Dual Lens Scope) placed inside the incubator to record activity levels on a scale of 1–4. We scored activity as follows: (1) Inactive—Beetle is inactive, either motionless with feet tucked in or standing still; (2) Low activity—Occasional movement of legs or antennae; slow, intermittent walking. (3) Medium activity—Continuous, steady walking within the chamber. (4) High activity—Rapid, erratic movement; beetle sprinting back and forth, sometimes attempting to fly. We recorded activity at the start of the 10-min acclimation period, during acclimation, at the beginning of the 5-min recording window, and continuously throughout the recording whenever the activity level changed. We recorded baseline CO_2_ readings at the beginning and end of each trial to account for any drift.

To process metabolic files, we used LoggerPro3 software. We corrected CO_2_ output values using equation (1) to account for flow rate (Lighton 2019).


(1) \begin{eqnarray*}
VC{O}_2 &=& FR\left( {F^{\prime}C{O}_2 - FC{O}_2} \right)/\nonumber\\
&&\left\{ {1 - F^{\prime}C{O}_2\left[ {1 - \left( {1/RQ} \right)} \right]} \right\}
\end{eqnarray*}

where $VC{O}_2$ is the rate of CO_2_ emission, *FR* is mass flow rate of air, $F^{\prime}C{O}_2$ is the fraction of CO₂ in expired air, $FC{O}_2$ is the fraction of CO₂ in inspired air, and *RQ* is the respiratory quotient. For RQ, we used the default value of 0.85. We selected the final CO_2_ outputs from within the 5-min recording window. To obtain as close to resting metabolic rates as possible, we divided the recording window into all possible continuous 60-s periods, selected the period with the lowest average CO_2_ production for each beetle, and then averaged activity across this 60-s period. Prior to data analysis, we excluded any beetles with mean activity levels equal to or greater than 3, which corresponded to medium and high activity. We also excluded beetles for which we did not have baseline measurements of CO_2_ production because we could not control for drift.

### Data analysis

To explore how metabolic rates varied across sexes and male morphs, we analyzed mean CO_2_ production using linear regression models (“lm” function in the “stats” package; R version 4.4.1) that included morph (major male, minor male, and female) as the primary predictor of interest. We included morph and its interaction with species as predictors since morph physiology could vary among the four species. The species in this study belong to one of two tribes (*Onthophagini* and *Phanaeini*). Analyzing these data using phylogenetically independent contrasts was not possible due to the lack of phylogenetic information (particularly for *O. hecate*). With only four species across two tribes, we could not account for relatedness via a mixed-effects model, since we would have too few random-effects levels to appropriately estimate the variability of the random effects of tribe or species. Moreover, within each tribe, CO_2_ production differs between sexes for one species but not the other (see the section “Results”). This suggests that differences in metabolic rates between morphs are not associated with relatedness.

Because body size can affect metabolic rates (Brown et al. 2004) and the relationship between body size and metabolic rate may vary by species (Fig. S2), we accounted for organism mass in our analyses following the recommendations of Lighton (2019). Specifically, we accounted for the power-law dependence of metabolic rate on beetle mass by log-transforming the response (mean CO_2_ output), including log-transformed beetle mass as a predictor in the regression model, and checking for any two-way and three-way interactions between log-transformed mass, morph, and species. We also ran a model without body mass to see if any differences we found were mediated by body mass or driven by intrinsic differences among morphs. We conducted separate analyses (see Supporting Methods) using an alternative approach to account for mass effects (i.e., mass-independent metabolic rates); however, regardless of the approach, we arrive at the same overall conclusions (Supporting Methods). In all models, we included mean activity level as a covariate since activity can impact metabolic rates. We tested for potential multicollinearity between our predictors of interest and other covariates in our model by evaluating $GVI{F}^{\frac{1}{{(2 \times df)}}}$ (where GVIF is the generalized variance inflation factor and *df* is the degrees of freedom of the predictor; Fox and Monette 1992). We computed these adjusted GVIFs using the full model without interactions via the “car” package in R (Fox and Weisberg 2019). The $GVI{F}^{\frac{1}{{(2 \times df)}}}$ of morph and species were under the threshold of 2.2 (values above 2.2 indicate high collinearity).

We found the three-way interaction among morph, species, and beetle mass was not supported. We removed the interaction between species and beetle mass since including this did not significantly improve the model [F(3,172) = 1.064, *P* = 0.3658]. Therefore, our final model of mean CO_2_ production included the effects of morph and species and their interaction, with beetle mass and mean activity levels as covariates.

We further investigated the morph by species interaction by conducting post-hoc pairwise comparisons of mean CO_2_ production between morphs for each species. To this end, we estimated and compared marginal means using the “emmeans” and “pairs” functions from the emmeans package in R (Lenth 2021), and corrected *P*-values for each family of comparisons by applying the “mvt” adjustment (based on the multivariate t distribution). We organized and visualized data using the R packages “dplyr” (Wickham et al. 2023) and “ggplot2” (Wickham 2016), respectively.

## Results

Across the four species, we analyzed data from 189 beetles, with a total of 54 females, 66 minor males, and 69 major males (Table 1). In all species, the average body mass of major males and females did not differ (mvt-adjusted pairwise comparisons of groups; *P* > 0.05), but the average body mass of minor males was significantly smaller than that of both major males and females (*P* < 0.05; Fig. S3, Fig. S4). Within two species, *P. vindex* and *O. hecate*, mean activity levels during the trials were similar on average across morphs (Table 1, Fig. S5). Minor males of *O. silenus* were less active on average than both females and major males of that species. Females of *O. taurus* also appeared less active during the trials than both major and minor males of the species.

**Fig. 1:**
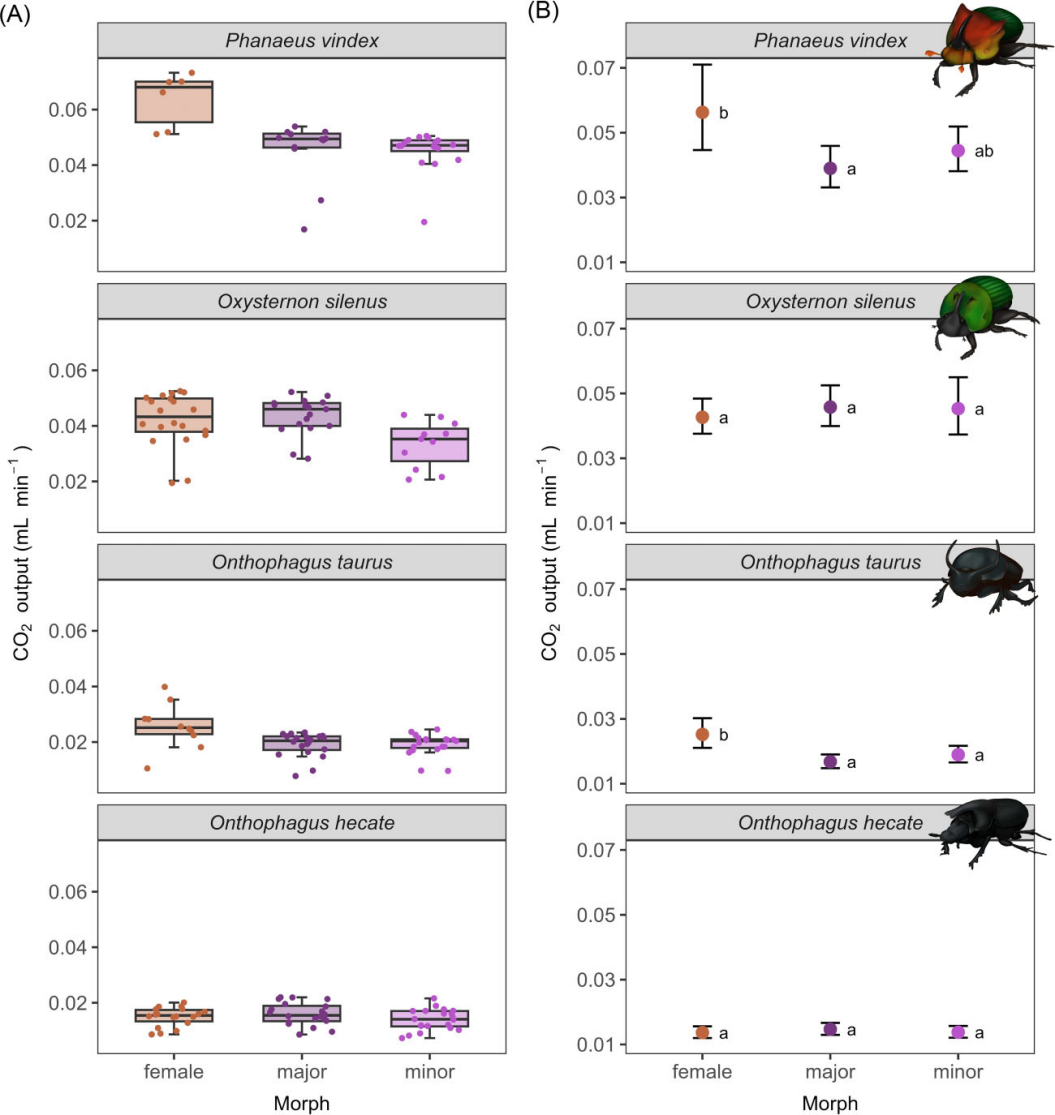
CO_2_ production for four species of dung beetle showing sexual and male dimorphism. (A) CO_2_ production for morphs of each species not accounting for beetle mass or activity. Boxplots correspond to the first and third quartiles, and whiskers extend to the most extreme data value within 1.5 times the interquartile range. Jittered points show data for individual beetles. (B) Expected CO_2_ production from beetles of a given morph with average mass for each species and average activity across species. Points show least squares means and 95% CIs around the means. Letter labels indicate significant differences.

Our goal was to determine whether the morphs (females, major males, and minor males) of dung beetle species differed in their metabolic rates. We predicted that females and minor males would have similar and higher metabolic rates than major males. Initially, we examined metabolic rates by building a model that did not account for beetle mass, but included morph, species, the interaction between morph and species, and activity. We found significant differences between the sexes only in *O. taurus* and *P. vindex* (Table S1), such that females had higher log CO_2_ production than males in both species (Fig. 1A).

Given that metabolic rate is highly impacted by body mass, we then investigated whether this effect was mediated by body mass or was entirely driven by intrinsic differences among morphs (Fig. 1B). Our final model, which explained 84.5% of the variation in log CO_2_ (Table S2), showed the following variables to be significant predictors of log CO_2_: species, the interaction between morph and species, log body mass, and mean activity (*P* < 0.05; Table 2). As expected, body mass and mean activity were major contributors to CO_2_ production: for every 10% increase in starting mass, expected mean CO_2_ production increases by 3.61%, and with every one-unit increase in activity level (within the range 1–2.8), CO_2_ production increases by a factor of 1.395, or 39.5% (Table S2). The significant interaction between morph and species suggests that whether and to what extent morphs differ in their CO_2_ production depends on the species of dung beetle.

**Table 2:** ANOVA results from the final best-fit model of log-transformed CO_2_ production as a function of morph, species, and the interaction between morph and species. The model also includes log-transformed individual mass (measured immediately preceding metabolic trials) and mean activity level as covariates.

Model term	Sum of squares	df	*F*	*P*
Morph	0.26168	2	2.502	0.08486
Species	0.60833	3	3.877	0.01024
log(Initial Mass)	0.55665	1	10.644	0.001328
Mean activity	3.24145	1	61.983	3.487e-13
Morph × Species	1.38791	6	4.423	0.0003432
Residuals	9.15179	175		

To further investigate the interaction, we conducted post-hoc pairwise tests to compare mean CO_2_ production between morphs of each species, after controlling for the effects of mass and activity level. We detected differences in CO_2_ production between sexes for *P. vindex* and *O. taurus* (adjusted *P* < 0.05; Table 3), but not for *O. hecate* and *O. silenus* (adjusted *P* > 0.05), and we found no differences in CO_2_ production between male morphs of any species (adjusted *P* > 0.68).

**Table 3:** Pairwise comparisons of estimated marginal means from a model of log CO_2_ production that includes effects of morph, species, the interaction between morph and species, log-transformed beetle mass, and activity level. Differences in CO_2_ production between the three morphs (females, major males, minor males) of each species are expressed as ratios of the geometric means for each group (on the response scale, mL min^−1^). t scores from significance tests compare each ratio against a value of 1, which represents the null hypothesis (i.e. no difference in CO_2_ production between groups). Lower and Upper CL refer to the lower and upper limits of the adjusted 95% confidence intervals. *P*-values and confidence intervals have been adjusted for multiple comparisons using the “mvt” method (using R package “emmeans”).

Species	Contrast	Ratio	SE	df	Lower CL	Upper CL	*T*	*P*
*O. hecate*	major/female	1.075	0.0811	175	0.866	1.334	0.959	0.9726
	minor/female	1.008	0.0771	175	0.809	1.254	0.099	1
	minor/major	0.937	0.0733	175	0.749	1.172	−0.829	0.9891
*O. silenus*	major/female	1.074	0.081	175	0.865	1.333	0.94	0.9758
	minor/female	1.062	0.1018	175	0.807	1.397	0.631	0.9983
	minor/major	0.989	0.096	175	0.749	1.306	−0.109	1
*O. taurus*	major/female	0.666	0.0611	175	0.512	0.866	−4.435	2e-04
	minor/female	0.752	0.073	175	0.569	0.993	−2.94	0.04
	minor/major	1.129	0.0868	175	0.906	1.407	1.58	0.6826
*P. vindex*	major/female	0.693	0.0794	175	0.499	0.962	−3.202	0.018
	minor/female	0.79	0.095	175	0.56	1.115	−1.958	0.4053
	minor/major	1.141	0.1104	175	0.864	1.505	1.359	0.8274

In *P. vindex*, both male morphs were associated with lower CO_2_ production than females. Adjusting for multiple comparisons, we found a significant difference between major males and females in CO_2_ production (adjusted *P* = 0.018; Table 3). Relative to females of the same mass and activity level, expected CO_2_ production in major males is 30.7% lower. While the expected CO_2_ production of minor males is 21% lower than that of females of the same mass and activity, this difference was not statistically significant (adjusted *P* = 0.405). The difference between the two male morphs was also not significant (adjusted *P* = 0.827), though CO_2_ production is expected to be 14.1% higher in minor males than in major males of the same mass and activity. Consequently, in *P. vindex*, we lack sufficient evidence to show that minor males differ from major males in CO_2_ production.

Similarly, in *O. taurus*, both male morphs were associated with lower CO_2_ production than females. Adjusting for multiple comparisons, we found significant differences in CO_2_ production both between major males and females (adjusted *P* = 0.0002) and between minor males and females (adjusted *P* = 0.04; Table 3). Relative to females of the same body mass and activity, we expect CO_2_ production to be 33.4% lower in major males and 24.8% lower in minor males, per our model. The difference between minors and majors was not significant (adjusted *P* = 0.683): expected CO_2_ production for a minor male is only 12.9% higher than for a major male of the same mass and activity level. Overall, for both *P. vindex* and *O. taurus*, the difference in CO_2_ production between the male morphs was found to be smaller than the difference in CO_2_ production between minor males and females (Fig. 1).

## Discussion

To understand how different developmental pathways and reproductive strategies shape energetics, we examined resting metabolic rates of four dung beetle species with both sexual and male dimorphism. We predicted that metabolic rates of females and minor males would be similar and higher than metabolic rates of major males. After accounting for activity levels and body size, we found a significant interaction between morph and species such that differences in CO_2_ production among morphs and the magnitude of those differences depend on the species of dung beetle. Of the four species we examined, metabolic rates of sexual morphs only differed in two species, *P. vindex* and *O. taurus*, with *P. vindex* showing significantly lower metabolic rates in major males compared to females, and *O. taurus* showing significantly lower metabolic rates in both major and minor males compared to females. We found no difference in metabolic rate among morphs in *O. hecate* and *O. silenus*, even when we did not account for body mass, indicating that these species have no detectable differences in energetic costs among morphs, at least based on resting metabolic rates. Despite showing alternative reproductive tactics, we found no significant difference in metabolic rates between major and minor morphs of any species.

Theoretical models of male alternative reproductive tactics usually separate well-provisioned competitive major males from the smaller, more poorly provisioned minor males that also invest more in sperm competition (Simmons et al. 1999). It has generally been assumed that building and maintaining large sexual weaponry, such as horns or swordtails, is energetically costly (Basolo and Alcarez 2003; Cummings and Gelineau-Kattner 2009; Garrison et al. 2025), but more recent theory integrating the allometry of sexually selected traits points out that mass-specific metabolic rates of larger individuals are lower than those of smaller individuals, which would result in positive allometry (Somjee 2021; Glazier 2024). In the stag beetle *Cyclommatus mniszechi*, body mass was a very strong predictor of standard metabolic rate in all morphs (females, major males, and minor males), but the slope of the relationship between body mass and standard metabolic rate was higher in the major males, suggesting that major males bore a significant energetic cost (Chen et al. 2024). By contrast, we found no interaction between body mass and morph in our work, suggesting that allometry did not differ across the morphs. However, research also shows that minor males of some species have elevated energetic costs associated with sperm traits. Though a majority of studies have not found significantly better sperm performance traits in sneaker compared to dominant males, studies that have found differences show sneaker males tend to have higher sperm performance trait values (reviewed in Kustra and Alonzo 2020; Simmons et al. 2007). Here we test these relationships across four species, finding no general trend in energetic costs across male morphs when body mass is either included or excluded, suggesting that energetic tradeoffs driving alternative reproductive tactics may occur in a species-specific manner and therefore are not likely the only reason for the existence of these tactics. Importantly, however, differences may have been observed if we had measured the metabolism of males during periods of greatest activity or in the presence of females (Cummings and Gelineau-Kattner 2009).

Females and males have different reproductive strategies that are expected to impact metabolic rates. Eggs are more energetically expensive to produce than sperm (Hayward and Gillooly 2011). Differences in the energetic costs of gamete production should result in higher metabolic rates in females than males, a pattern that has been observed in some insects, including *Drosophila melanogaster* (Klok et al. 2010; Wat et al. 2020) and bed bugs (DeVries et al. 2014), and a pattern we have shown here in the dung beetles *O. taurus* and *P. vindex*. Yet, we found no sex-specific differences in metabolic rates in either *O. silenus* or *O. hecate* dung beetles. This may be due to other energetic costs associated with male reproductive physiology and behavior. For example, in 12 species of seed beetle (Bruchinae), males generally had a higher metabolic rate than females (Arnqvist et al. 2022), which could be related to male investment in ejaculate biomass production since males produce seminal fluid in addition to gametes (Immonen et al. 2016; Arnqvist et al. 2022). Similarly, males of both field crickets and their dipteran parasitoids showed higher metabolic rates than females. This may be due to male crickets and fly parasitoids participating in costly mating behaviors, such as mate-searching or courtship activity (Kolluru et al. 2004). In dung beetles, the production of seminal fluid and/or mating behaviors of males could increase energetic costs of reproduction, making energetic costs of reproduction in males relatively similar to those in females. Indeed, previous work on seed beetles has shown a negative correlation in the evolution of mating costs in males and females (i.e., as mating costs increase in one sex, they tend to decrease in the other). Regardless of the mechanism, differences in metabolic rates between the sexes vary significantly among dung beetle species and other insects (e.g., Arnqvist et al. 2022), with no clear trends between males and females.

Here, we found no general trend in energetic costs among dung beetles showing sexual and male dimorphism. This indicates that the energetic tradeoffs related to development and reproductive strategies may be species-specific, and therefore models that assume a particular sex or male morph has higher energetic costs should be re-evaluated. Going forward, investigating a broader range of potential explanations for male alternative reproductive tactics than just energetic tradeoffs may provide more robust hypotheses.

## Supplementary Material

obaf031_Supplemental_Files

## Data Availability

The data underlying this article are available in the online supplementary material.
