# Supplementary material for: Testing for Differences in Metabolism Among Females and Dimorphic Males of Four Dung Beetle Species (Coloeoptera: Scarabaeinae)
Source: Integr Org Biol. 2025 Jul 26;7(1):obaf031. doi: 10.1093/iob/obaf031 (PMC12392089; doi:10.1093/iob/obaf031)
Supplement: obaf031_Supplemental_Files [file obaf031_supplemental_files.zip › Supplementary Figures and Tables.pdf]

Supplementary Figures and Tables

Testing for differences in metabolism among females and dimorphic males of four dung beetle species (Coloeoptera: Scarabaeinae)

Alexander T. Killeffer, J. Morgan Fleming, Anchal Padukone, Nathan Duerr, Katherine A. Reed, Jonier Merizalde-Toro, Katie E. Marshall, Jorge E. Celi, Kimberly S. Sheldon

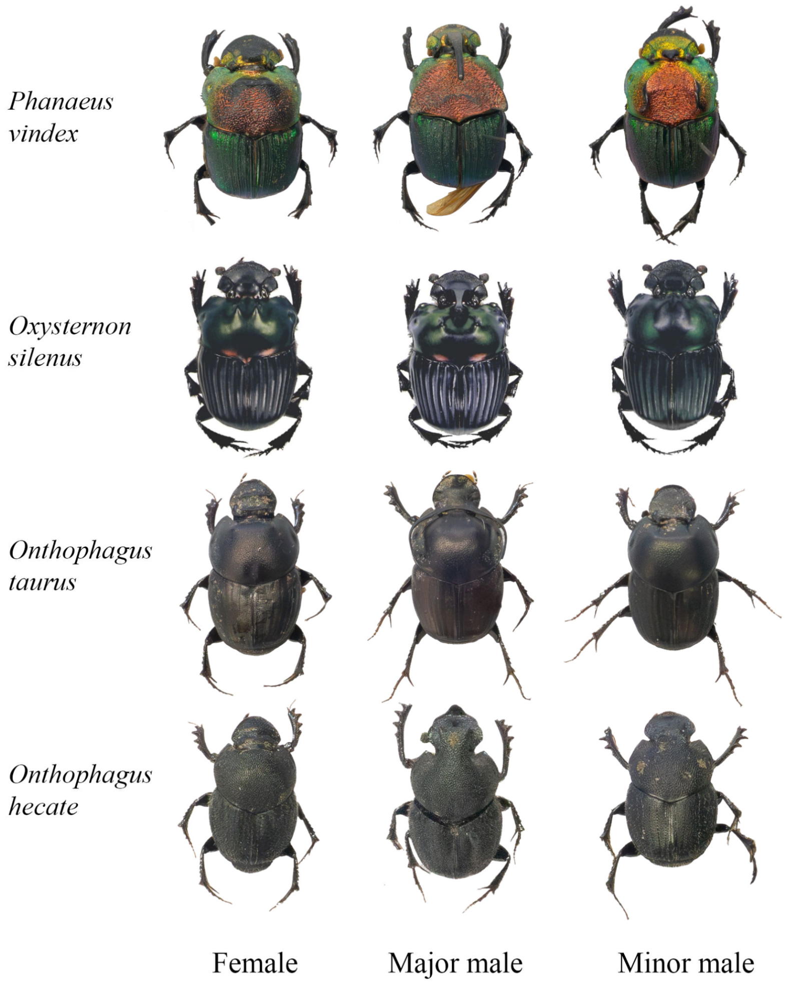

Figure S1. Photos of study species by morph. Images of Phanaeus vindex, Onthophagus taurus, and Onthophagus hecate are from beetles we captured in Tennessee, USA. Images of Oxysternon silenus from Peru are from Edmonds and Zedík (2004) and used with permission.

A)

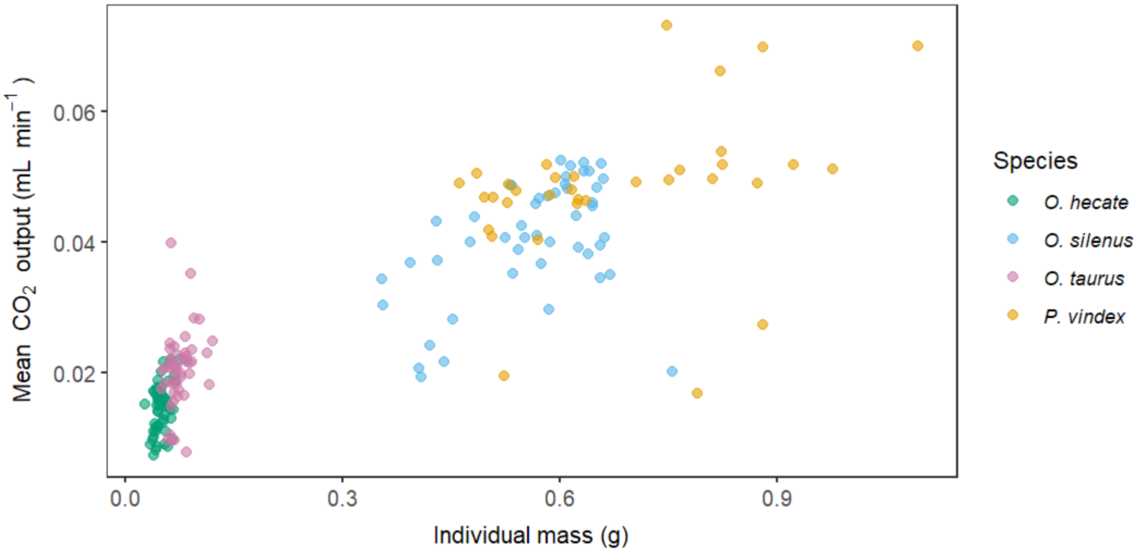

B)

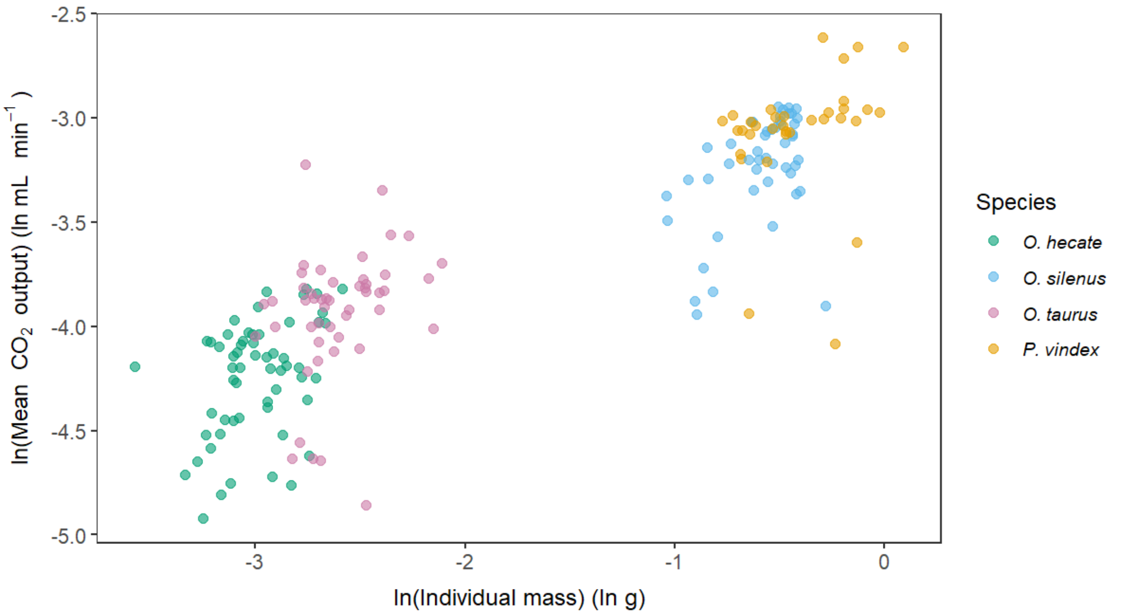

Figure S2: A) Relationship between beetle mass (g) and mean CO_2 production (mL min^{-1}). B) Relationship between log mass (ln g) and log mean CO_2 output (ln mL min^{-1}).

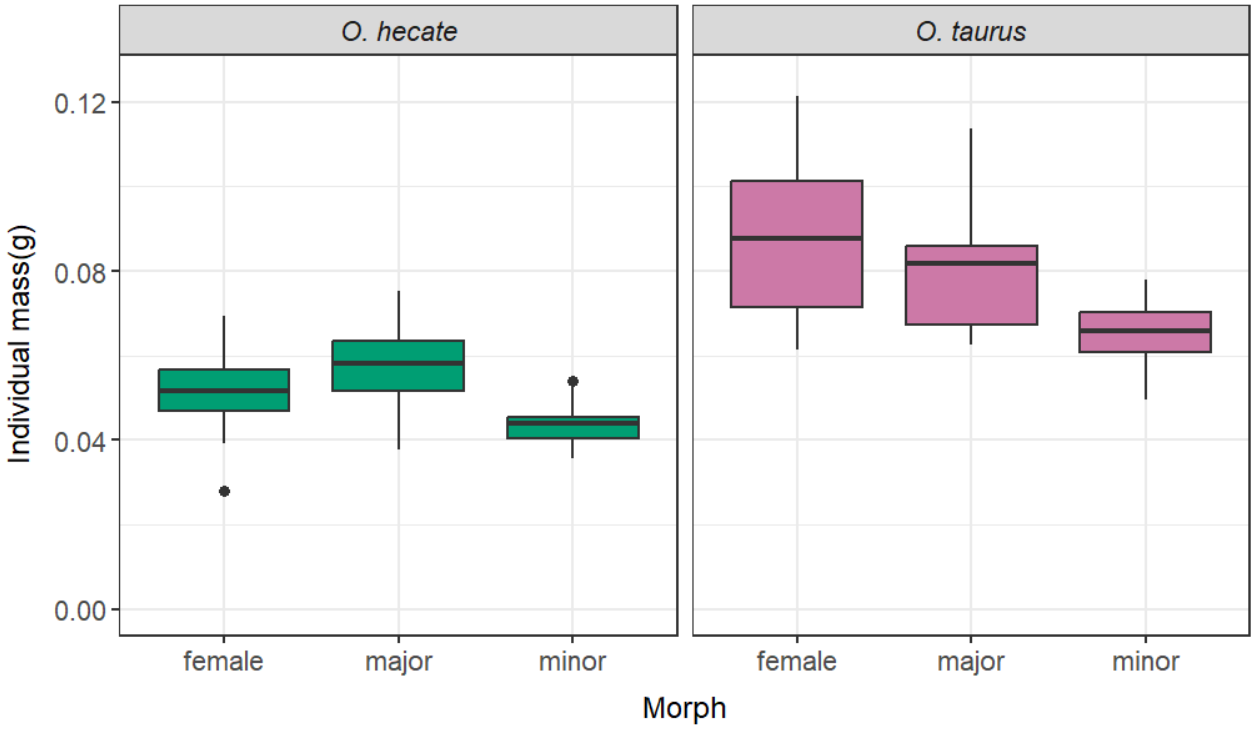

Figure S3: Mass (g) of *O. hecate* and *O. taurus* beetles (the two smaller species in the study), grouped by morph. Boxes represent the inter-quartile range for each group, with the dark line indicating the median. Solid circles indicate mass values more than 1.5 times the inter-quartile range away from the box. For both species, the average mass of minor males is significantly lower than the average mass of major males and that of females (mvt-adjusted pairwise comparisons of group means; $p < 0.05$).

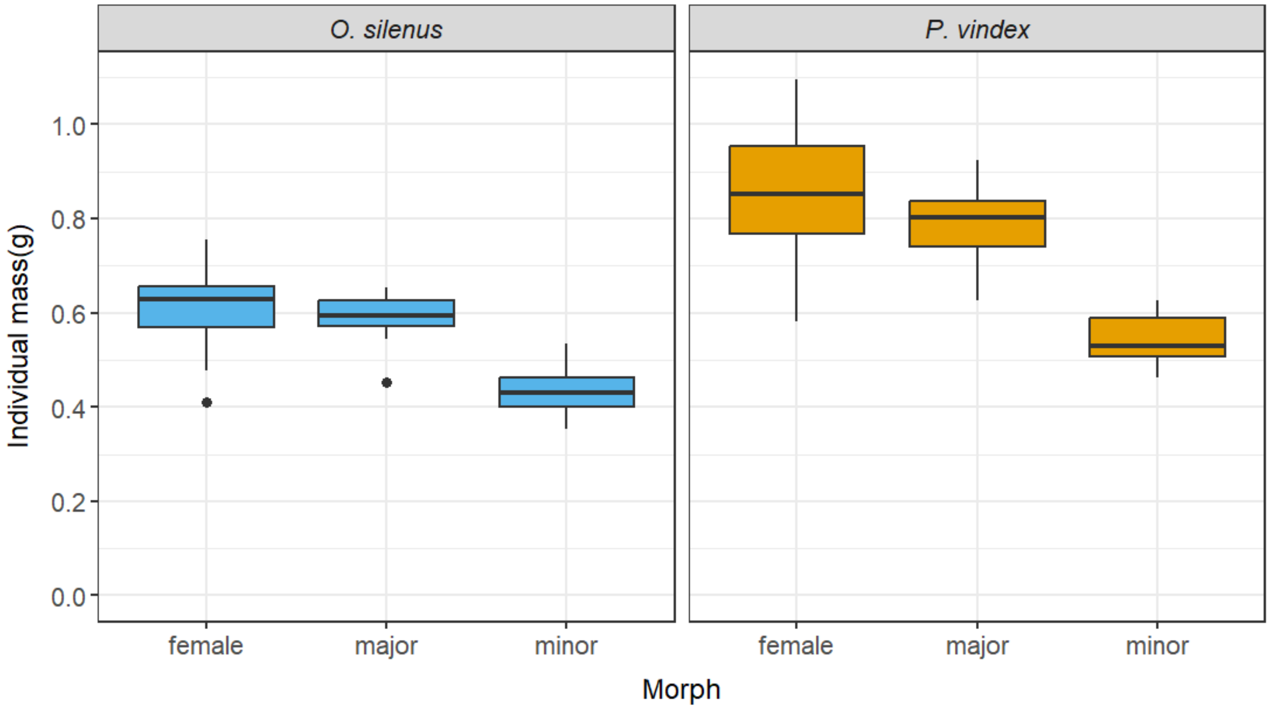

Figure S4: Mass (g) of *O. silenus* and *P. vindex* beetles (the two larger species in the study), grouped bymorph. Boxes represent the inter-quartile range for each group, with the dark line indicating the median. Solid circles indicate mass values more than 1.5 times the inter-quartile range away from the box. For both species, the average mass of minor males is significantly lower than the average mass of major males and that of females (mvt-adjusted pairwise comparisons of group means; $p < 0.05$).

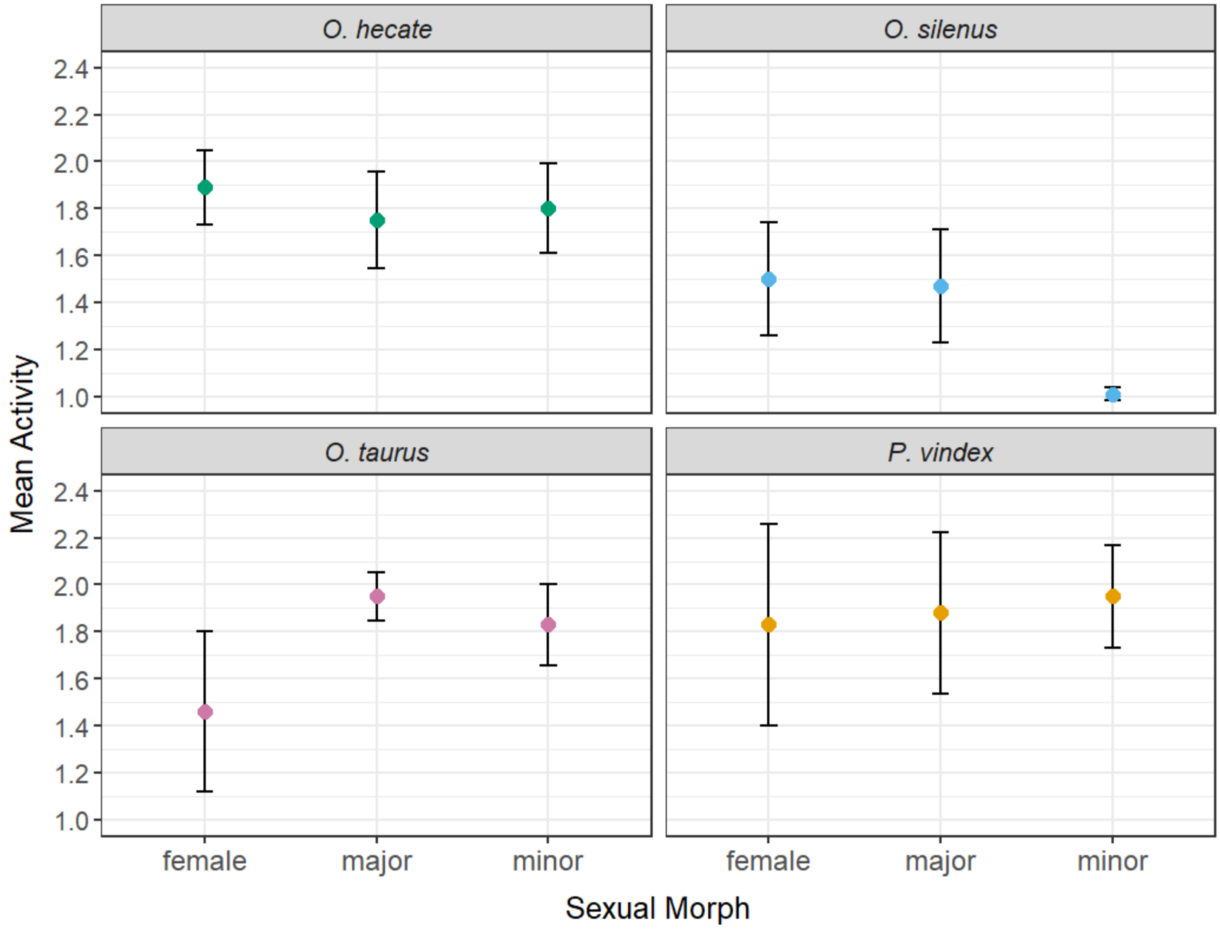

Figure S5: Mean activity levels of different species grouped by morph. Filled circles indicate mean activity level for each morph and the error bars indicate 95% confidence intervals of the mean.

Table S2. Pairwise comparisons of estimated marginal means from a model of log CO_2 production that includes effects of morph, species, activity level, and the interaction between morph and species, but excludes the effect of beetle mass. Differences in CO_2 production between the morphs of each species are expressed as ratios of the geometric means for each group (on the response scale, mL min^{-1}). *t* scores from significance tests compare each ratio against a value of 1, which represents the null hypothesis (i.e. no difference in CO_2 production between groups). Lower and Upper CL refer to the lower and upper limits of the adjusted 95% confidence intervals. *P*-values and confidence intervals have been adjusted for multiple comparisons using the "mvt" method (using R package "emmeans").

**Species**	**Contrast**	**Ratio**	**SE**	**df**	**Lower CL**	**Upper CL**	***t***	***P***
O. hecate	major / female	1.117	0.0855	176	0.897	1.391	1.450	0.7768
	minor / female	0.950	0.0726	176	0.764	1.183	-0.665	0.9977
	minor / major	0.851	0.0632	176	0.687	1.052	-2.178	0.2724
O. silenus	major / female	1.065	0.0826	176	0.853	1.330	0.819	0.9904
	minor / female	0.941	0.0853	176	0.726	1.220	-0.671	0.9975
	minor / major	0.883	0.0821	176	0.677	1.153	-1.337	0.8439
O. taurus	**major / female**	0.640	0.0598	176	0.489	0.836	-4.784	**4.32e-05**
	**minor / female**	0.671	0.0625	176	0.514	0.876	-4.285	**0.0004**
	minor / major	1.049	0.0791	176	0.845	1.302	0.632	0.9984
P. vindex	**major / female**	0.675	0.0793	176	0.482	0.945	-3.346	**0.0113**
	**minor / female**	0.672	0.0756	176	0.487	0.928	-3.530	**0.0060**
	minor / major	0.996	0.0894	176	0.770	1.288	-0.049	1

Table S3. Regression summary for the final best-fit linear model of log-transformed CO_2 production as a function of morph, species, and the interaction between morph and species. The model also includes log-transformed beetle mass (measured immediately preceding metabolic trials) and mean activity level as covariates.

Model term	Parameter estimate	Standard error	t-statistic	*p*
(Intercept)	-3.75672	0.35669	-10.532	2.116e-20
Morph (major)	0.07241	0.07547	0.959	0.3387
Morph (minor)	0.00759	0.07652	0.099	0.9211
Species (*O. silenus*)	0.24503	0.29242	0.838	0.4032
Species (*O. taurus*)	0.46701	0.11035	4.232	3.731e-05
Species (*P. vindex*)	0.44993	0.33668	1.336	0.1832
log(Initial Mass)	0.37219	0.11408	3.263	0.001328
Mean activity	0.33311	0.04231	7.873	3.487e-13
Morph (major): Species (*O. silenus*)	-0.00143	0.10692	-0.013	0.9894
Morph (minor): Species (*O. silenus*)	0.05283	0.11638	0.454	0.6505
Morph (major): Species (*O. taurus*)	-0.47945	0.12106	-3.96	0.0001087
Morph (minor): Species (*O. taurus*)	-0.29323	0.11902	-2.464	0.01472
Morph (major): Species (*P. vindex*)	-0.43948	0.13802	-3.184	0.001719
Morph (minor): Species (*P. vindex*)	-0.24315	0.13633	-1.784	0.07623

Multiple R^2 = 0.845
Residual standard error: 0.2287 on 175 degrees of freedom
