# Supplementary material for: Testing for Differences in Metabolism Among Females and Dimorphic Males of Four Dung Beetle Species (Coloeoptera: Scarabaeinae)
Source: Integr Org Biol. 2025 Jul 26;7(1):obaf031. doi: 10.1093/iob/obaf031 (PMC12392089; doi:10.1093/iob/obaf031)
Supplement: obaf031_Supplemental_Files [file obaf031_supplemental_files.zip › Supplementary Methods.pdf]

Supplementary Methods

Testing for differences in metabolism among females and dimorphic males of four dung beetle species (Coloeoptera: Scarabaeinae)

Alexander T. Killeffer, J. Morgan Fleming, Anchal Padukone, Nathan Duerr, Katherine A. Reed, Jonier Merizalde-Toro, Katie E. Marshall, Jorge E. Celi, Kimberly S. Sheldon

Accounting for the relationship between organismal mass and metabolic rate

We accounted for organism mass in our analyses following the recommendations of Lighton (2008). Specifically, we accounted for the power-law dependence of metabolic rate on beetle mass by log-transforming the response (mean CO_2 output), including log-transformed beetle mass as a predictor in the regression model, and checking for any two-way and three-way interactions between log-transformed mass, morph and species. Since including the interactions between mass and morph/species did not significantly improve the model, our final model predicted log-transformed CO_2 output as a function of species, morph and their interaction, log-transformed mass and activity level. This approach (i.e. including mass as a covariate in an ANCOVA/regression model) enables us to compare metabolic rate across groups while holding mass and activity level constant (e.g. Chown et al. 2016; Carter and Sheldon 2020).

Other studies obtain "mass-independent metabolic rates" by regressing metabolic rate on body mass and extracting the residuals (Cameron and Marshall 2024) or by correcting the metabolic rate for mass using allometric equations. We arrive at the same overall conclusions whether we take the former or the latter approach to accounting for beetle mass. In supplementary analyses (detailed below), we regressed (1) log-transformed metabolic rate on log-transformed body mass using data from all species and obtained the residuals; (2) log-transformed metabolic rate on log-transformed body mass separately for each species and obtained the residuals. The scaling relationships between CO_2 production and mass for all organisms in this study and for each species are given in Table 1 on the following page.

We then analyzed each set of mass-corrected CO_2 production rates simply as a function of species, morph and their interaction, and activity level. Results from analyses of variance and pairwise comparisons between morphs are given in: (1) Tables 2 and 4 (response = mass-corrected metabolic rates using overall scaling relationship); (2) Tables 3 and 5 (response = mass-corrected metabolic rates using species-specific scaling relationships).

Either approach to mass-correcting CO_2 output revealed a statistically significant interactive effect of species and morph on metabolic rate (Tables 2 and 3). Further investigating this interaction via post hoc comparisons between morphs revealed that *P. vindex* females had significantly higher mass-corrected CO_2 production rates relative to *P. vindex* major males and that *O. taurus* females had higher mass-corrected CO_2 production rates relative to both male morphs of this species (Tables 4 and 5).

Table 1: Scaling relationships between live body mass (M, g) and metabolic rate (MR, mL min^{-1}) for the four dung beetle species in the study.

**Species**	**Allometric equation**
Onthophagus hecate	$MR = 0.0735 \times M^{0.5444}$
Oxysternon silenus	$MR = 0.0592 \times M^{0.6893}$
Onthophagus taurus	$MR = 0.0573 \times M^{0.4127}$
Phanaeus vindex	$MR = 0.0522 \times M^{0.2726}$
Overall	$MR = 0.0530 \times M^{0.4167}$

Table 2: ANOVA results from the model of mass-corrected log-CO_2 output (corrected for mass using the overall scaling relationship) as a function of morph, species, activity level and the interaction between morph and species.

**Model term**	**Sum of squares**	**df**	***F***	***p***
Morph	0.25816	2	2.480	0.08664
**Species**	0.70098	3	4.490	**0.00460**
**Mean activity**	3.23349	1	62.130	**3.22959e-13**
**Morph × Species**	1.38653	6	4.440	**0.00033**
Residuals	9.15974	176		

Table 3: ANOVA results from the model of mass-corrected log-CO_2 output (corrected for mass using species-specific scaling relationships) as a function of morph, species, activity level and the interaction between morph and species.

**Model term**	**Sum of squares**	**df**	***F***	***p***
Morph	0.26017	2	2.524	0.08300
Species	0.40768	3	2.637	0.05122
**Mean activity**	3.24252	1	62.927	**2.39639e-13**
**Morph × Species**	1.49262	6	4.828	**0.00014**
Residuals	9.06904	176		

Table 4: Pairwise comparisons of mass-corrected log-CO_2 output (corrected using the overall scaling relationship) between morphs for each species. Differences are expressed as the ratio of the geometric means for each group (on the response scale). *t* scores from significance tests compare each ratio against a value of 1, which represents the null hypothesis (i.e. no difference in CO_2 output between groups). Lower and Upper CL refer to the lower and upper limits of the adjusted 95% confidence intervals. p-values and confidence intervals have been adjusted for multiple comparisons using the "mvt" method (using R package "emmeans").

**Species**	**Contrast**	**Ratio**	**SE**	**df**	**Lower CL**	**Upper CL**	***t***	***p***
O. hecate	major / female	1.070	0.0796	176	0.865	1.324	0.912	0.9809
	minor / female	1.015	0.0753	176	0.820	1.255	0.196	1
	minor / major	0.948	0.0684	176	0.771	1.166	-0.737	0.9952
O. silenus	major / female	1.075	0.0809	176	0.866	1.333	0.955	0.9746
	minor / female	1.078	0.0949	176	0.837	1.387	0.851	0.9877
	minor / major	1.003	0.0906	176	0.774	1.299	0.034	1
O. taurus	**major / female**	0.669	0.0607	176	0.516	0.867	-4.434	**2e-04**
	**minor / female**	0.762	0.0689	176	0.588	0.987	-3.007	**0.0330**
	minor / major	1.139	0.0834	176	0.923	1.405	1.778	0.5409
P. vindex	**major / female**	0.695	0.0793	176	0.501	0.964	-3.190	**0.0187**
	minor / female	0.806	0.0881	176	0.589	1.102	-1.978	0.3963
	minor / major	1.159	0.1011	176	0.903	1.488	1.695	0.6031

Table 5: Pairwise comparisons of mass-corrected log-CO_2 output (corrected using species-specific scaling relationships) between morphs for each species. Differences are expressed as the ratio of the geometric means for each group (on the response scale). *t* scores from significance tests compare each ratio against a value of 1, which represents the null hypothesis (i.e. no difference in CO_2 output between groups). Lower and Upper CL refer to the lower and upper limits of the adjusted 95% confidence intervals. p-values and confidence intervals have been adjusted for multiple comparisons using the "mvt" method (using R package "emmeans").

**Species**	**Contrast**	**Ratio**	**SE**	**df**	**Lower CL**	**Upper CL**	***t***	***p***
O. hecate	major / female	1.056	0.0782	176	0.855	1.306	0.743	0.9950
	minor / female	1.035	0.0765	176	0.838	1.279	0.472	0.9998
	minor / major	0.980	0.0704	176	0.798	1.204	-0.280	1.0000
O. silenus	major / female	1.081	0.0809	176	0.872	1.339	1.036	0.9588
	minor / female	1.181	0.1035	176	0.919	1.518	1.899	0.4514
	minor / major	1.093	0.0982	176	0.845	1.414	0.988	0.9687
O. taurus	**major / female**	0.668	0.0603	176	0.516	0.866	-4.462	**2e-04**
	**minor / female**	0.761	0.0685	176	0.588	0.985	-3.037	**0.0302**
	minor / major	1.138	0.0830	176	0.924	1.403	1.776	0.5419
P. vindex	**major / female**	0.688	0.0781	176	0.497	0.953	-3.294	**0.0134**
	minor / female	0.757	0.0823	176	0.554	1.034	-2.561	0.1136
	minor / major	1.100	0.0954	176	0.858	1.410	1.099	0.9425

References

Cameron H, Marshall D. 2024. Estimating the relationship between fitness and metabolic rate: which rate should we use? *Philosophical Transactions of the Royal Society B: Biological Sciences* 379:20220491.

Carter AW, Sheldon KS. 2020. Life stages differ in plasticity to temperature fluctuations and uniquely contribute to adult phenotype in *Onthophagus taurus* dung beetles. *Journal of Experimental Biology* 223.

Chown SL, Haupt TM, Sinclair BJ. 2016. Similar metabolic rate-temperature relationships after acclimation at constant and fluctuating temperatures in caterpillars of a sub-Antarctic moth. *Journal of Insect Physiology* 85:10–16.
